# Supplementary material for: Model states for a class of chiral topological order interfaces
Source: Nat Commun. 2019 Apr 23;10:1861. doi: 10.1038/s41467-019-09168-z (PMC6478732; doi:10.1038/s41467-019-09168-z)
Supplement: Supplementary file 1 — Supplementary Information [file 41467_2019_9168_MOESM1_ESM.pdf]

Supplementary Information: Model States for a Class of Chiral Topological Order Interfaces

V. Crépel[1], N. Claussen[1], B. Estienne[2] and N. Regnault[1]
[1] *Laboratoire de Physique de l'École Normale supérieure, ENS, Université PSL, CNRS, Sorbonne Université, Université Paris Diderot, Sorbonne Paris Cité, Paris, France and*
[2] *Sorbonne Université, CNRS, Laboratoire de Physique Théorique et Hautes Énergies, LPTHE, F-75005 Paris, France*

We provide here additional details about the convergence of the numerically computed quantities discussed in the main text. We also show some important checks bolstering our results.

Supplementary Note I. Finite Size Effects

As stated in the main text, we exploit the discrete translation symmetry mapping one orbital to another on the cylinder geometry to obtain an iMPS description of the Laughlin 1/2 and Halperin 221 states. While the system becomes infinite in the x-direction, the cylinder perimeter L introduces finite size effects. The thermodynamic limit is reached only when L becomes much larger than the bulk correlation length. The bulk Laughlin and Halperin correlation lengths have been computed numerically[1,2] and are slightly smaller than the magnetic length. Thus finite size effects are of no concern in the bulks for the system size considered in the paper ($L \simeq 10-12\ell_B$). The spin-resolved densities, shown for different perimeters in Supplementary Fig. 1, are indeed identical for any perimeters larger than $10\ell_B$.

However, some ripples may be seen close to the interface for the leftmost plot of Supplementary Fig. 1, corresponding to the data used in the main text. The two other graphs show that the ripples observed at the transition quickly disappear with the cylinder perimeter increases and are thus interpreted as finite size effects. Though present in our simulations, we mitigate these finite size effects by only considering objects twice or three times greater than the bulks correlation lengths. Because of the entanglement area law[3], it often requires to consider a MPS bond dimension increasing exponentially with the surface of the objects considered. Recall that the MPS auxiliary space is the CFT Hilbert space of a two-component free boson, which we truncate with respect to conformal dimension. In the following, we denote as $P_{\max}$ the truncation parameter (see Ref.[4] for a precise definition). $P_{\max}$ behaves as a logarithmic measure of the bond dimension. The main numerical limitation then comes from the truncation of the auxiliary space and the saturation effects that we now investigate as a function of $P_{\max}$. Note that the density is a robust quantity that quickly converges with respect to the truncation parameter $P_{\max}$. Thus, we can safely consider larger perimeters, as in Supplementary Fig. 1 which was obtained for $P_{\max} = 10$.

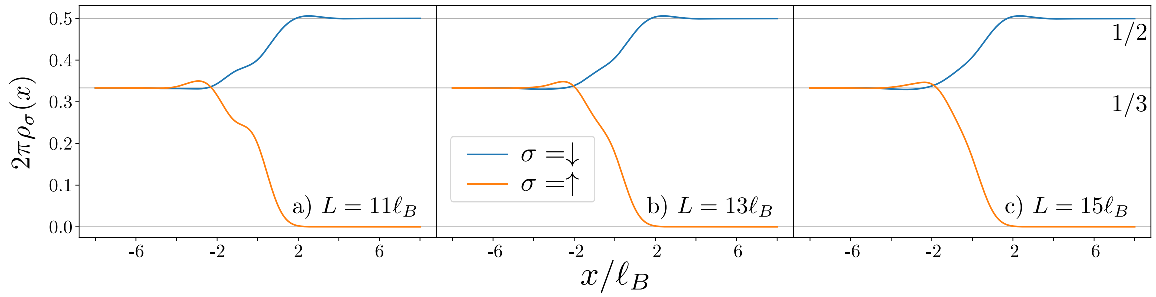

Supplementary Fig. 1. *Spin resolved densities across the transition for different cylinder perimeters L. The ripples at the transition quickly disappear when the cylinder perimeter L increases and are thus interpreted as finite size effects.*

Supplementary Note II. Rotationally Invariant Cut

To fix the notations, we choose the origin for the x-axis between the Laughlin and Halperin orbitals, *i.e.* the center of the LLL orbitals for which Laughlin (resp. Halperin) iMPS matrices are used are $x_n = \left(\frac{2\pi}{L}\right)(n+1/2)\ell_B^2$ (resp. $x_n = -\left(\frac{2\pi}{L}\right)(n+1/2)\ell_B^2$) with $n \in \mathbb{N}$. We consider a rotationally symmetric bipartition with $\mathcal{A} = \{(x',y')|x' < x, 0 \leq y' \leq L\}$. We numerically compute the RSES and the corresponding Von Neumann EE $S_{\mathcal{A}}(L,x)$ for various cylinder perimeters L. As claimed in the main text, we always observe an area law behavior for which the first correction is constant

$$S_{\mathcal{A}}(L,x) = \alpha(x)L - \gamma(x) \tag{1}$$

This linear behavior is well satisfied numerically as shown for a cut at $x = 0$ in Figs. 2a. In Supplementary Fig. 2b and c, we compare the results of a linear regression of $S_{\mathcal{A}}(L,x)$ with respect to L with the numerical evaluation of $\alpha(x) = \partial_L S_{\mathcal{A}}(L,x)$ and $\gamma(x) = S_{\mathcal{A}}(L,x) - L\partial_L S_{\mathcal{A}}(L,x)$ with finite differences. The corrections to these constants are of subleading order, and are much more sensitive to finite size effects ($L \leq 9\ell_B$ on Supplementary Fig. 2) or to saturation effects due to the truncation of the auxiliary space ($L \geq 14\ell_B$ on Supplementary Fig. 2).

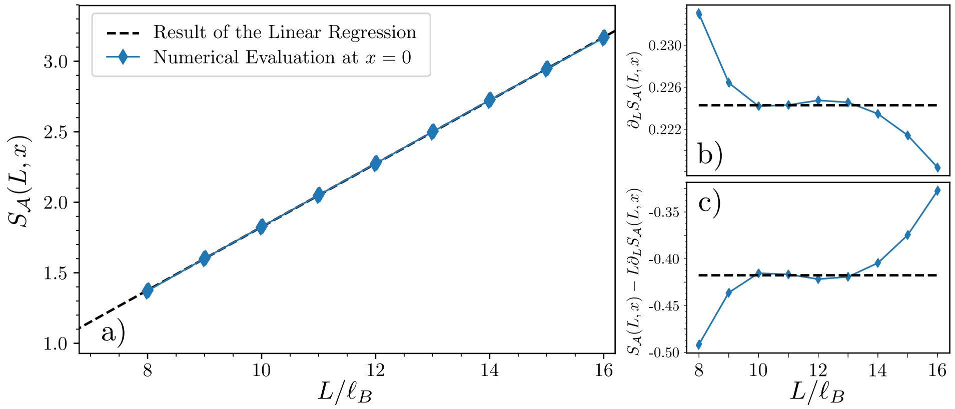

Supplementary Fig. 2. *a) The EE $S_{\mathcal{A}}(L, x)$ shows perfect agreement with the area law of Supplementary Eq. (1) – here illustrated for $x = 0$ and computed for a truncation parameter $P_{\max} = 12$. Comparaison between the numerical evaluation of b) $\alpha(x) = \partial_L S_{\mathcal{A}}(L, x)$ and c) $\gamma(x) = S_{\mathcal{A}}(L, x) - L\partial_L S_{\mathcal{A}}(L, x)$ with finite differences and the results of the linear regression. Both agree over the range of perimeters $L \sim 11 - 13\ell_B$ where both finite size effects and saturation effects due to the finite bond dimension are controlled.*

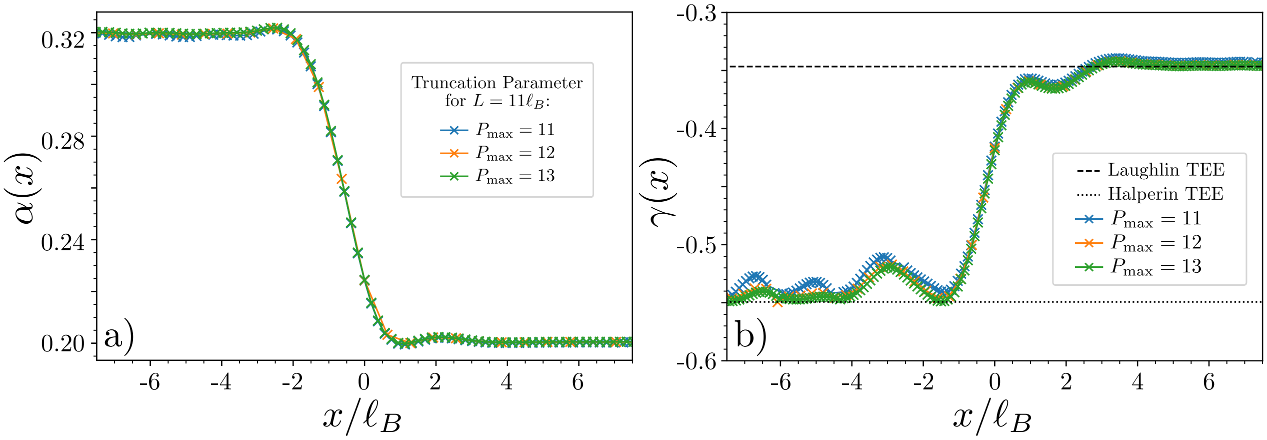

Supplementary Fig. 3. *Convergence of the numerically extracted a) $\alpha(x)$ and b) $\gamma(x)$ at $L = 11\ell_B$ with respect to the truncation parameter $P_{\max}$. As expected, the subleading term $\gamma(x)$ is more affected by the saturation effects, especially in the region where $\alpha(x)$ is large. This occurs on the Halperin side, i.e. $x < 0$. Note that b) reproduces the results shown in the main text.*

The latter point is easily understood when noticing that in order to faithfully describe a state satisfying Supplementary Eq. (1), the bond dimension χ of the MPS should grow exponentially with the cylinder perimeter $\chi \sim e^{\alpha(x)L}$. However, we notice that in the range $L \simeq 11 - 13\ell_B$, we can reliably use either techniques (fit or discrete derivative) to compute the coefficients $\alpha(x)$ and $\gamma(x)$. These are the perimeters we extensively use in the main text and thereafter.

In particular, we focus on $L = 11\ell_B$ and we study the convergence of the numerically extracted $\alpha(x)$ and $\gamma(x)$ near the transition. Supplementary Fig. 3 shows the numerical results for $x \in [-7.5, 7.5]$ (we refer to Ref.[2] for a similar analysis deep in the bulks). We observe that for greater $\alpha(x)$ (for instance in the Halperin region) $\gamma(x)$ is more sensitive to saturation effects, as expected from our previous discussion. Note that the computation for $P_{\max} = 13$ requires an auxiliary space of dimension $\chi = 41\,558$.

Supplementary Note III. Levin-Wen Subtraction Scheme

A. Covering Entirely the Gapless Mode

We first assert that the identification of a $c = 1$ theory at the interface does not depend on finely tuned parameters. The choice of the cylinder perimeter $L = 12\ell_B$ is only motivated by the avoidance of finite size effects, as seen on

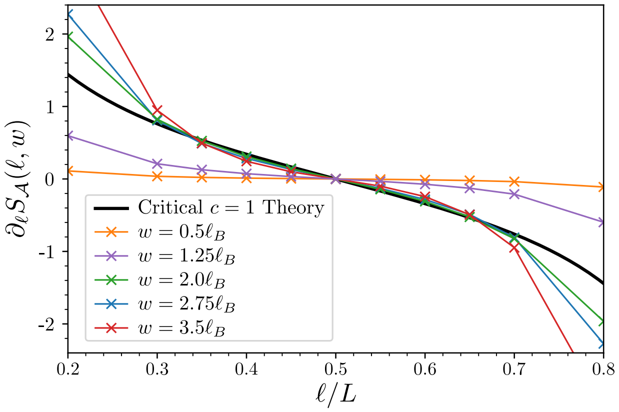

Supplementary Fig. 4. *Derivative of the EE computed with the Levin Wen scheme described in the text for different width of the patch across the interface. For $w \in \{0.5, 1.25, 2.0, 2.75, 3.5\}$, the fitted central charge are respectively 0.04, 0.26, 1.03, 0.99, 1.08. Either the patch is large enough to entirely cover the critical mode $w \geq 2\ell_B$ and the central charge extracted is close to one, or it misses completely or part of the critical mode and we find $c < 1$. This analysis quantitatively shows that the central charge estimation provided in the main text does not come from a fine tuned choice of our patch.*

Fig. 3 in the main text. Depending on x_1 and x_2, the patch may however not fully cover the critical mode and the extracted value of c be a fraction of the real central charge of the underlying interface theory. To investigate this, we varied the width of the patch over a few magnetic lengths. For each value of w depicted in Supplementary Fig. 4, we fit the central charge with the previously described method. We observe that the fitted central charge increases (respectively 0.04 and 0.26 for $w = 0.5\ell_B$ and $w = 1.25\ell_B$) to reach a constant value around one for w large enough (respectively 1.03, 0.99 and 1.08 for $w =\in \{2, 2.75, 3.5\}$). This reliably point toward a critical theory of central charge $c = 1$, which is only entirely captured when the patch is wide enough to fully cover the gapless interface mode. Note that higher values of w are plagued by the finite auxiliary space dimension. Increasing w leads to a higher area law contribution along x, requiring an even larger truncation parameter $P_{\max}$. The first artifacts of such saturation effects are seen for $w = 3.5\ell_B$ in Supplementary Fig. 4 and are investigated further in the next section.

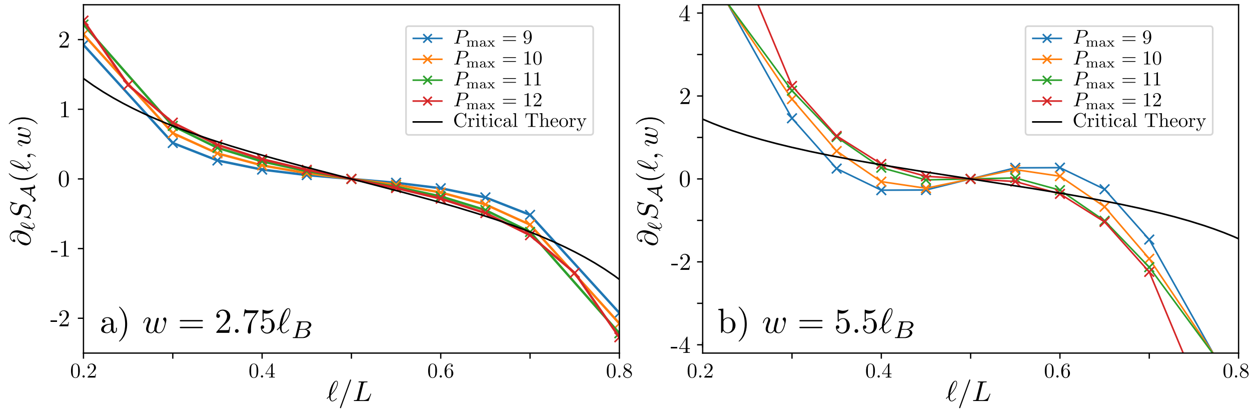

Supplementary Fig. 5. *Convergence of the derivative of the EE with respect to the truncation parameter $P_{\max}$. a) When w is small, the contribution of the critical mode at the interface to the EE may be extracted when the area law contribution are minimal (here for $w = 2.75\ell_B$). b) Saturation effects dominates when the width w is increased. Indeed, it leads to a higher area law contribution along x, requiring an even larger truncation parameter $P_{\max}$ (here for for $w = 5.5\ell_B$).*

B. Truncation Effects

To exemplify saturation effects, we consider two patches of respective width $w = 2.75\ell_B$ and $w = 5.5\ell_B$. The numerical analysis described in the main text is performed and the results are presented in Supplementary Fig. 5. The bond dimension required to faithfully capture the entanglement properties, especially the subleading logarithmic

term coming from the gapless mode at the interface, grows exponentially with the width of the patch (see discussion above). We indeed see that when $w = 2.75\ell_B$ the EE converges to the theoretical prediction of Eq. (5) with increasing truncation parameter P_{max}, up to finite size effects when the patch is smaller than or comparable to the bulks correlation lengths. When $w = 5.5\ell_B$, the area law contributions in the x direction are not fully accommodated by the finite auxiliary space and saturation effects dominate. Hence, the EE for finite auxiliary spaces and large patches deviates from Eq. (5), even if the trend seems to indicate that it will converge to the expected behavior with increasing P_{max}.

[1] B. Estienne, N. Regnault, and B. A. Bernevig, Phys. Rev. Lett. **114**, 186801 (2015).
[2] V. Crépel, B. Estienne, B. A. Bernevig, P. Lecheminant, and N. Regnault, Phys. Rev. B **97**, 165136 (2018).
[3] N. Laflorencie, Phys. Rep. **646**, 1 (2016).
[4] B. Estienne, N. Regnault, and B. A. Bernevig, Preprint at arXiv:1311.2936 [cond-mat.str-el].
